# Development of a Juglone/Mesoporous Carbon Modified Glassy Carbon Electrode for Ultrasensitive Determination of Melatonin in Dietary Supplements

**DOI:** 10.3390/nano16160979

**Published:** 2026-08-10

**Authors:** Joanna Smajdor-Baran, Katarzyna Fendrych

**Affiliations:** Department of Analytical Chemistry and Biochemistry, Faculty of Materials Science and Ceramics, AGH University of Krakow, Al. A. Mickiewicza 30, 30-059 Krakow, Poland

**Keywords:** juglone, melatonin, mesoporous carbon, modified electrode, voltammetry

## Abstract

A novel electrochemical sensing platform based on a glassy carbon electrode (GCE) modified with a juglone/mesoporous carbon composite (JUG-MC/GCE) was developed for the ultrasensitive determination of melatonin (MEL) in pharmaceutical formulations and dietary supplements. Incorporation of juglone and mesoporous carbon within the coating deposited on the glassy carbon electrode, resulted in enhanced charge-transfer characteristics at the electrode–electrolyte interface. Under optimized differential pulse voltammetry (DPV) conditions in a 0.1 mol L^−1^ McIlvaine buffer (pH 2.8), the proposed sensor demonstrated an exceptional electrocatalytic response for melatonin oxidation via a two-electron, one-proton irreversible pathway under kinetic control. The analytical performance reveal multiple linear calibration intervals with an ultra-low limit of detection (LOD) of 0.21 µg L^−1^ (0.9 nM). The sensor manifested robust operational repeatability (RSD ≤ 1.8%), long-term storage stability, and satisfactory selectivity toward melatonin in the presence of common tablet excipients and selected potential interferents. The practical utility of the JUG-MC/GCE platform was successfully validated through the direct quantification of melatonin in commercial tablets, capsules, and complex jelly matrices, confirming its suitability for melatonin determination in real samples.

## 1. Introduction

Melatonin (N-acetyl-5-methoxytryptamine) is an endogenous indoleamine hormone synthesized predominantly by the pineal gland [1,2]. It serves as a master regulator of the mammalian circadian clock, orchestrating sleep–wake cycles, modulating immune responses, and acting as a potent endogenous free-radical scavenger within antioxidant defense networks [3,4,5,6,7]. Given its multifaceted physiological significance and expanding therapeutic applications, the precise quantification of melatonin (MEL) in pharmaceutical formulations, complex biological matrices, and dietary supplements has become a focal point of contemporary analytical research. To date, conventional benchmarking techniques, predominantly high-performance liquid chromatography (HPLC) [8,9], liquid chromatography-mass spectrometry (LC-MS) [10,11,12], and various spectrophotometric assays [13,14,15], deliver excellent sensitivity and low limits of detection. Nevertheless, these methodologies are frequently bottlenecked by the requirement for capital-intensive instrumentation, laborious and multi-step sample preparation protocols, high consumption of hazardous organic solvents, and prolonged analysis times.

To address the limitations associated with conventional analytical techniques, electrochemical sensing strategies have emerged as highly compelling alternatives, owing to its inherent simplicity, rapid multi-sample throughput, cost-effectiveness, and compatibility with decentralized, onsite miniaturization. Because the analytical performance of electrochemical sensors and overpotential of an electrochemical assay are intrinsically dictated by the interfacial architecture of the working electrode, engineering novel surface modifiers remains a paramount objective in electroanalysis. Specifically, research focuses on developing advanced materials capable of accelerating heterogeneous electron transfer kinetics, mitigating surface fouling, and expanding the electroactive surface area, thereby improving sensitivity and analytical reliability [16,17,18].

Recent years have witnessed considerable progress in the development of electrochemical sensors for melatonin determination base on advanced sensing materials [19,20,21,22,23,24,25,26,27,28,29,30,31,32]. Nevertheless, many of the proposed approaches rely on sophisticated multicomponent nanocomposites, molecular imprinting strategies, or multistep fabrication procedures, which may increase preparation time, complexity, and cost. Therefore, there remains a need for simple, robust, and cost-effective sensing platforms capable of providing comparable analytical performance while facilitating practical implementation for routine analysis of melatonin-containing pharmaceutical formulations and dietary supplements.

In this context, structured mesoporous carbon (MC) materials have attracted profound interest as electrocatalytic scaffolds. Their popularity stems from an interconnected network of well-defined pores, exceptionally high specific surface areas, superb intrinsic electrical conductivity, and chemical resilience [33,34,35,36,37,38,39]. These structural hallmarks not only facilitate unhindered mass transport of the analyte via rapid intrapore diffusion but also provide a dense distribution of accessible active sites for localized adsorption. Consequently, MC frameworks consistently yield amplified current responses and depressed detection limits. Furthermore, capitalizing on host-guest chemistry by anchoring redox-active organic mediators within these carbonaceous matrices represents a powerful strategy to induce electrocatalytic synergy at the electrode-electrolyte interface.

Juglone (5-hydroxy-1,4-naphthalenedione), a naturally occurring phenolic alpha-quinone, stands out as an exemplary candidate for such surface modification due to its highly reversible, proton-coupled redox behavior and superb electron-mediating kinetics [40,41,42,43]. When immobilized, its quinone/hydroquinone couple can act as an effective electrocatalytic relay. The strategic combination of mesoporous carbon with juglone (JUG) is hypothesized to create a highly conductive microenvironment that lowers the activation energy required for melatonin oxidation, thereby drastically enhancing signal transduction and minimizing oxidative overpotentials.

Herein, we report the fabrication of a novel electrochemical sensing platform utilizing a mesoporous carbon-juglone composite matrix for the sensitive determination of melatonin. The interfacial charge-transfer dynamics and electrochemical traits of the modified electrode were systematically interrogated via voltammetry and electrochemical impedance spectroscopy (EIS). Furthermore, the analytical merit of the juglone/mesoporous carbon modified glassy carbon electrode (JUG-MC/GCE) was rigorously evaluated across critical performance indicators, including linear dynamic range, limit of detection, reproducibility, and selectivity against common interferents. Finally, the practical utility of the platform was validated through the routine assay of melatonin in commercial dosage forms, highlighting the broader viability of quinone-functionalized carbon composites in modern electroanalysis.

## 2. Experimental

### 2.1. Chemicals and Solution

All chemicals utilized in this study were of analytical grade and required no further purification. Melatonin (98%) was obtained from Chemat (Gdansk, Poland), while juglone (5-hydroxy-1,4-naphthalenedione, 97%) was sourced from AmBeed (Buffalo Grove, IL, USA). Polystyrene M_w_∼35,000) and mesoporous carbon (<500 nm particle according to the manufacturer’s specification) were both procured from Sigma Aldrich (St. Louis, MO, USA). Dichloromethane (ACS reagent) and acetone (HPLC grade, ≥99.9%) were purchased from Honeywell Research Chemicals (Seelze, Germany) and Sigma-Aldrich, respectively. To prepare the 1000 mg L^−1^ melatonin (MEL) stock solution, the compound was dissolved gravimetrically in ethanol and double-distilled water (*v*/*v* 1:1) and kept at 4 °C; working standards of lower concentrations were freshly prepared each day. Additionally, a 0.1 mol L^−1^ citrate-phosphate buffer (McIlvaine buffer) solution with pH from 2.4 to 4.0 was prepared by mixing appropriate volume of 0.1 mol L^−1^ citric acid with 0.2 mol L^−1^ Na_2_HPO_4_ (Avantor Performance Materials Poland S.A, Gliwice, Poland). Tested interferents, i.e., caffeine, ascorbic acid, cellulose, starch, magnesium stearate, and titanium dioxide, were purchased from Sigma Aldrich, while citric acid, glucose, and uric acid were obtained from Avantor.

### 2.2. Instrumentation

The particle size distribution (PSD) of the dispersed mesoporous carbon was determined by laser diffraction using a Malvern Mastersizer 2000 (Malvern Instruments, Malvern, UK). The measurements were performed in aqueous dispersion, and the particle size distribution was calculated using the General Purpose analysis model. Scanning electron microscopy (SEM) was employed to investigate the surface morphology of the juglone, mesoporous carbon, and the modified electrode layer. The observations were carried out using a FEI Nova NanoSEM 200 microscope (Thermo Fisher Scientific, Waltham, MA, USA).

Electrochemical assessments were carried out using an M161 multipurpose electrochemical analyzer with an M164 electrode stand (mtm-anko, Krakow, Poland), with signal processing and data management handled via EALab (v. 2.1), Origin 2023b, and Matlab R2026a. Voltammetric trials utilized a standard three-electrode arrangement consisting of a JUG-MC/GCE working electrode (3 mm glassy carbon diameter, BASi, West Lafayette, IN, USA), a Mineral (Gliwice, Poland) Ag|AgCl (3 mol L^−1^ KCl) reference, and a platinum wire counter electrode. Agitation at ~200 rpm was maintained using a Kartell (Noviglio, Italy) Teflon^®^-coated magnetic stir bar. Prior to negative potential measurements, solutions were deoxygenated with argon. Buffer pH adjustments were monitored with a SevenCompact S210 pH meter (Mettler Toledo, Greifensee, Switzerland).

The electrochemical impedance spectroscopy (EIS) investigations were conducted on a Metrohm Autolab system (Eco Chemie AUT32N.FRA2, Herisau, Switzerland) running NOVA software. The cell assembly for EIS comprised a bare GCE or the corresponding modified electrode, a 3 mol L^−1^ Ag|AgCl|KCl reference, and a platinum rod counter electrode. Impedance spectra were acquired in a mixture of 2 mmol L^−1^ K_4_[Fe(CN)_6_] and 0.1 mol L^−1^ KCl. The DC bias potential was set individually for each electrode to the formal potential of the redox probe, experimentally determined from the corresponding cyclic voltammograms, namely 0.138 V for GCE, 0.400 V for JUG/GCE, 0.450 V for MC/GCE, and 0.327 V for JUG-MC/GCE. A sinusoidal AC perturbation with an amplitude of 10 mV RMS was applied over a frequency range from 1 MHz to 10 mHz.

### 2.3. Electrode Preparation

To prepare the modifying suspension, equal amounts (50 mg) of juglone and mesoporous carbon were blended and thoroughly homogenized using a mortar and pestle. Next, a 10 mg portion of this composite was transferred to an Eppendorf tube and mixed with 5 mg of polystyrene. The mixture was dissolved in a solvent system containing 600 μL of acetone and 400 μL of dichloromethane. After 40 min of homogenization using a Biosan (Riga, Latvia) vortex mixer, the suspension was kept in a refrigerator. Before application, the mixture underwent 15 min of agitation. The final step involved drop-casting a 10 μL aliquot of the suspension onto a GCE, which had been pre-polished to a mirror-like finish with 0.3 μm Al_2_O_3_ alumina powder (Buehler Micropolish II, Lake Bluff, IL, USA). To evaluate the contribution of the individual components, control electrodes based on JUG or MC were prepared following the same procedure described above. Specifically, 10 μL of a suspension containing 10 mg of either JUG or MC dispersed with the polymer binder was deposited onto the GCE surface, resulting in the juglone-modified GCE (JUG/GCE) and mesoporous carbon-modified GCE (MC/GCE), respectively.

### 2.4. Voltammetric Procedures

The electrochemical behavior of MEL on the JUG-MC/GCE platform was evaluated through Cyclic Voltammetry (CV) and Differential Pulse Voltammetry (DPV). While CV served to elucidate the specific oxidation pathways and redox mechanics of melatonin at the modified electrode, DPV was implemented to achieve highly sensitive quantitative measurements. All voltammetric data were collected in a 0.1 mol L^−1^ McIlvaine buffer at pH 2.8, utilizing a potential range between 400 and 1000 mV. To probe the kinetics of the MEL oxidation process, CV scan rates were adjusted across a spectrum from 6.3 to 500 mV s^−1^. The fine-tuned DPV parameters consisted of a 5 mV step potential (*E*_s_), a 50 mV pulse amplitude (*dE*), alongside waiting (*t*_w_) and sampling (*t*_s_) periods of 10 ms and 10 ms, respectively. Furthermore, a 20-s equilibration time was enforced between sequential runs to guarantee signal reproducibility. Prior to each analytical measurement, a DPV curve of the supporting electrolyte (blank solution) was recorded under identical experimental conditions. The background current was subsequently removed by subtracting the blank voltammogram from the voltammogram recorded in the presence of melatonin. The resulting background-corrected curves were used for peak current evaluation and calibration.

Quantification of MEL in the pharmaceutical formulation and dietary supplements was achieved using the standard addition method. Initially, DP voltammograms were registered for the blank solution, consisting of 5 mL of a 0.1 mol L^−1^ McIlvaine buffer (pH 2.8) in the voltammetric cell. Measurements were then performed following the addition of the diluted samples, as well as after three consecutive spikes of a 10 mg L^−1^ MEL standard solution. For every step of the analytical process, four successive scans were registered. Background correction was executed by subtracting the baseline signal of the supporting electrolyte measured prior to the addition of the electroactive species. The final concentration of MEL within the investigated samples was computed from the generated standard addition calibration curve, adjusting for the respective dilution factors.

### 2.5. Sample Preparation

#### 2.5.1. Tablets

Commercially available melatonin pharmaceutical tablets (3 mg per tablet) and dietary supplement tablets (1 mg per tablet) were purchased from a local pharmacy. For each formulation, five tablets were randomly selected, weighed, and finely ground into a homogeneous powder using an agate mortar. To prepare the stock solutions of pharmaceutical and dietary supplement an adequate portion of powder were weighted and transferred to obtain the final concentration of 60 and 10 mg L^−1^, respectively. Dissolution was carried out using a 1:1 (*v*/*v*) mixture of ethanol and double-distilled water. Both mixtures were subjected to ultrasonic homogenization for 15 min to ensure complete dissolution, followed by filtration through a 0.45 μm mixed cellulose ester (MCE) syringe filter (Biosense, Warszawa, Poland). Immediately prior to the DPV assays, a 10-fold dilution for the filtered pharmaceutical solution was performed using the supporting electrolyte.

#### 2.5.2. Capsules

A dietary supplement formulated as capsules, each containing 1 mg of melatonin, was evaluated. To ensure a uniform sample, the contents of five individual capsules were combined in a mortar and thoroughly homogenized by grinding. From the blended powder, an aliquot equivalent to 1 mg of melatonin was weighed and transferred to a 10 mL volumetric flask and filled with solvent mixture of ethanol and double-distilled water (*v*/*v* 1:1). To facilitate complete dissolution and extraction, the suspension was subjected to ultrasonic homogenization for 15 min. The resulting extract was then passed through a 0.45 µm MCE syringe filter to remove particulate matter. Just before conducting the voltammetric measurements, the filtered stock solution was diluted by a factor of 10.

#### 2.5.3. Jelly Candies

For the analysis of the jelly candies formulation (labelled content: 1 mg of melatonin per jelly), three whole jellies were randomly selected, weighed, and transferred directly into an amber glass 50 mL volumetric flask. Dissolution was carried out using a 1:1 (*v*/*v*) mixture of ethanol and double-distilled water. To ensure complete degradation of the hydrocolloid matrix and quantitative extraction of melatonin, the mixture was subjected to ultrasonic homogenization for 20 min at an elevated temperature (approx. 40 °C), followed by magnetic stirring until the jellies were completely dissolved. After cooling to room temperature, the flask was filled to the mark with the solvent mixture and filtered through a 0.45 μm MCE syringe filter. Immediately prior to the voltammetric assays, a 10-fold dilution of the filtered solution was performed to achieve a final nominal concentration of 6 mg L^−1^.

## 3. Results and Discussion

### 3.1. Characterization of Modifiers

**Figure 1:**
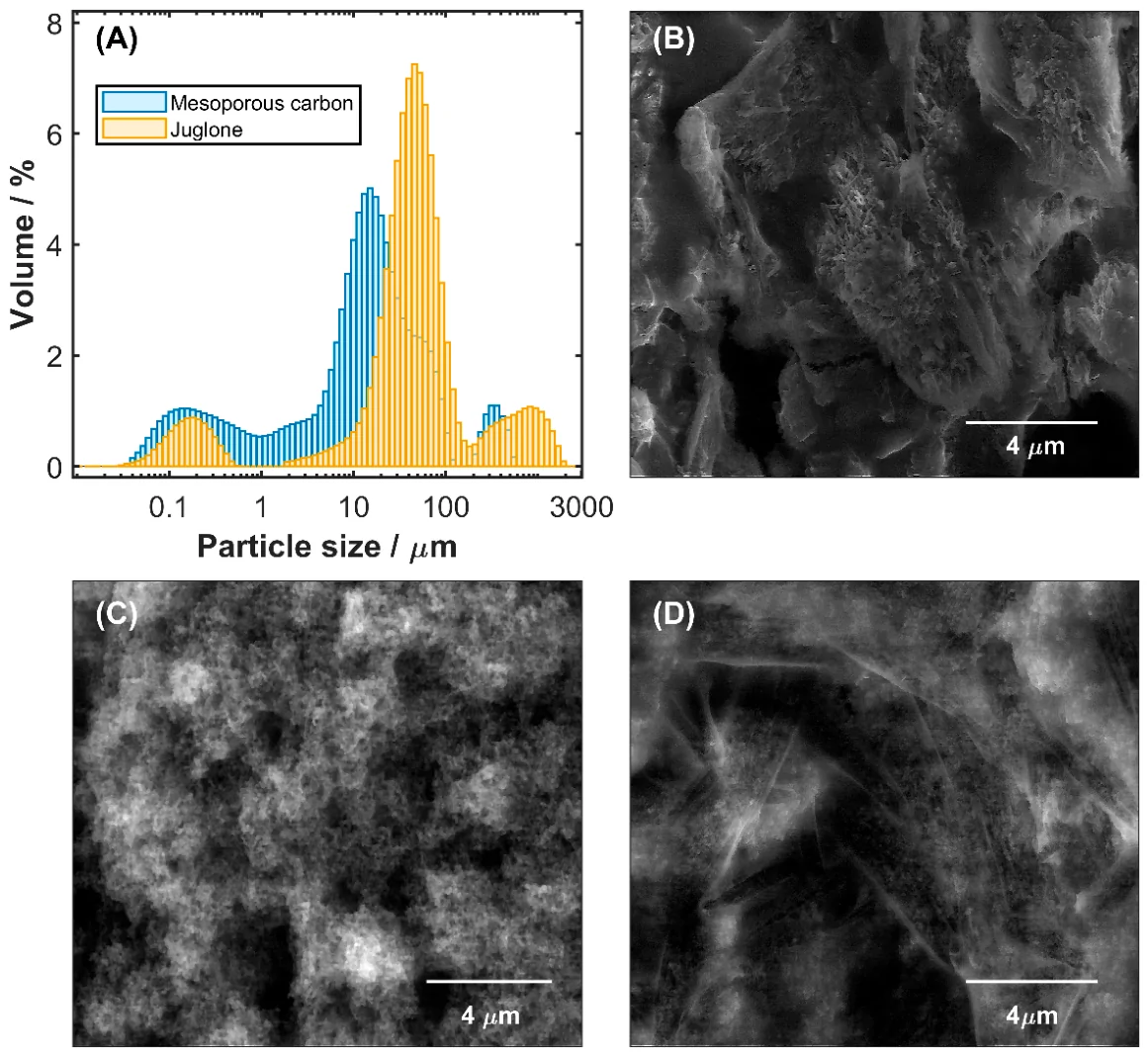
Material characterization. (**A**) Particle size distribution of juglone and mesoporous carbon particles/agglomerates used for GCE modification. SEM image of (**B**) juglone, (**C**) mesoporous carbon, and (**D**) juglone/mesoporous carbon modifying layer.

The modifiers used for GCE modification, mesoporous carbon and juglone, were evaluated to characterize their dispersion behavior prior to electrode fabrication. As can be seen in Figure 1A, both materials exhibit broad and multimodal particle size distributions, indicating the presence of fractions with different characteristic dimensions.

The distribution obtained for MC is dominated by micrometer-sized particles, with the main population occurring int the range of approximately 5–50 µm and a maximum contribution at around 15–20 µm. Additionally, a presence of minor fraction of particles below 1 µm and larger agglomerated structures exceeding 200 µm was observed. It should be noted that the measured particle size corresponds to the external dimensions of dispersed particles/agglomerates, which may differ from the size of individual carbon domains and does not directly reflect the mesoporous structure of MC. The latter is related to the internal pore architecture of the carbon material.

In contrast, juglone exhibits a particle size distribution shifted towards larger particle dimensions, with the predominant fraction located in the range of approximately 40–60 µm. Similar to mesoporous carbon, a small fraction of fine particles below 1 µm and a secondary population of particles in the several hundred micrometer range are present. The differences in particle size distribution between MC and JUG may influence their dispersion within the composite modification layer and consequently affect the morphology and electrochemical response of the modified electrode.

The SEM image of juglone (Figure 1B) shows irregularly shaped particles aggregated into larger clusters. The material exhibits a heterogeneous morphology characterized by rough surfaces, layered structures, and numerous surface irregularities. Plate-like and flake-like features with dimensions in the micrometer range can be observed, reflecting the characteristic morphology of the JUG particles. In the SEM image of mesoporous carbon (Figure 1C), a heterogeneous morphology composed of interconnected carbon particles forming irregular aggregates can be distinguished. The material exhibits a rough surface morphology with numerous cavities and irregularly distributed voids on the micrometer and submicrometer scales. In contrast to JUG, no distinct plate-like crystalline structures are observed. Instead, the carbon appears as interconnected aggregates creating a sponge-like architecture, characteristics for carbon-based porous materials. Finally, the SEM image of the juglone/mesoporous carbon composite layer (Figure 1D) reveals a compact and relatively surface morphology. The distinct morphological features of the individual components are less pronounced after composite formation, indicating the formation of a mixed carbon/juglone coating. The observed morphology suggesting a close physical association between JUG and MC within the modifier layer. The successful preparation of the composite electrode was further evaluated based on its electrochemical performance and comparison with control electrodes.

### 3.2. Electrochemical Characteristics of Modified Electrodes

**Figure 2:**
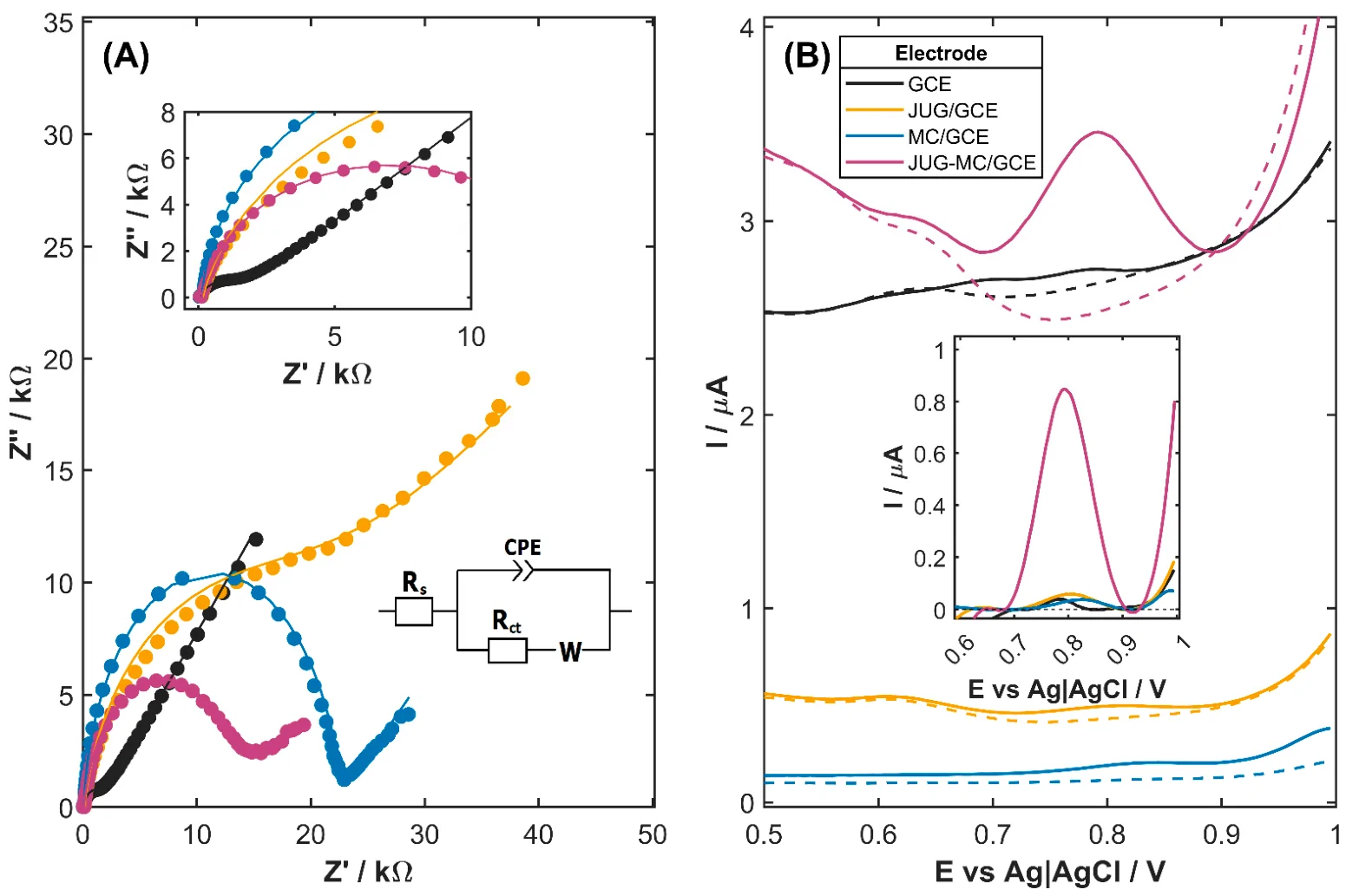
(**A**) The impedance spectra recorded for 2 mmol L^−1^ K_4_[Fe(CN)_6_] in 0.1 mol L^−1^ KCl solution on the investigates electrodes (symbols) and corresponding fitted spectra (solid lines). Inset: Randles cell equivalent circuit. (**B**) Comparison of DP voltammograms recorded for 0.2 mg L^−1^ MEL on the GCE, JUG/GCE, MC/GCE, and JUG-MC/GCE. Dashed lines represent the background signal. Inset: DP voltammograms subjected to the background correction. Experimental conditions: *E*_p_ = 0.4 V, *E_k_* = 1.0 V, *E_s_* = 5 mV, *dE* = 50 mV, *t*_w_ = 10 ms, *t*_s_ = 10 ms. Supporting electrolyte: 0.1 mol L^−1^ McIlvaine buffer solution (pH 2.8).

The influence of the individual components of the modifying layer on the electrochemical behavior of the JUG/GCE, MC/GCE, and JUG-MC/GCE electrodes was investigated using electrochemical impedance spectroscopy (EIS) (Figure 2A). The measurements were conducted in triplicate in 0.1 mol L^−1^ KCl solution containing 2 mmol L^−1^ [Fe(CN)_6_]^4−^, which served as a model redox probe. The experimental impedance spectra, presented as Nyquist plots, were fitted using the equivalent circuit model of a standard Randles cell (inset in Figure 2A). The non-ideal capacitive behavior of the electrode/electrolyte interface was represented by a constant phase element (CPE), characterized by the modulus (*Q*) and exponent of the constant phase element (*q*). Based on the fitted parameters, the charge transfer resistance (*R*_ct_) and Warburg impedance parameter (σ), were calculated according to Equations (1) and (2) [44,45]:

(1) Rct=RTn2F2Aelks·2(DODR)14DO12·CO+DR12·CR

(2) σ=RTn2F2Ael2·4DO12·CO+DR12·CR

where *R* indicate the universal gas constant (*R* = 8.314 J mol^−1^ K^−1^), *T*—the temperature [K], *F*—the Faraday constant (*F* = 96,485.3 C mol^−1^), *D*_O_ and *D*_R_—the diffusion coefficients of the oxidized and reduced forms, respectively, and *C*_O_ and *C*_R_ are their concentrations in the solution.

Dividing Equation (1) by Equation (2) provides the formula for the heterogeneous rate constant (*k*_s_), which is expressed as follows:

(3) $$k_{s}=\frac{\sigma }{R_{ct}}\cdot \sqrt{\frac{D_{O/R}}{2}}$$

where *D*_O_/_R_ means the average diffusion coefficient of the oxidized and reduced forms.

By converting the Formula (2), the electrochemically active surface area (*A*_el_) of the electrode to be calculated based on the determined value of σ, according to the Formula (4):

(4) Ael= RTn2F2σ2·4DO12·CO + DR12·CR

The electrical double-layer capacitance (*C*_dl_), normalized by the electrochemically active electrode area, was calculated from the fitted constant phase element (CPE) parameters (*Q* and *q*) according to Equation (5) [46]:

(5) Cdl=(Q·Rct1−q)1qAel

The fitting quality was assessed by calculating the residuals between the experimental and fitted impedance values. No pronounced systematic deviations were observed, indicating that the proposed equivalent circuits adequately described the experimental impedance responses. The quality of the equivalent-circuit fitting was quantitatively evaluated using the root mean square error (RMSE), calculated separately for the real ($Z^{'}$) and imaginary ($-Z^{\prime \prime }$) components of the impedance (Table 1). The obtained goodness-of-fit parameters indicate that the proposed equivalent circuits adequately described the experimental impedance spectra.

The Nyquist plot obtained for the bare GCE (Figure 2A) is characterized by a small high-frequency semicircle followed by a straight line in the low-frequency region. The small diameter of the semicircle correspond to a low charge transfer resistance (*R*_ct_ = 1.38 ± 0.02 kΩ, Table 1), indicating minimal kinetic limitation at the electrode/electrolyte interface. The nearly linear low-frequency segment corresponds to the Warburg impedance, confirming that the electrochemical process is partially controlled by the diffusion of electroactive species. Consequently, the bare GCE serves as an appropriate reference electrode for evaluating the effect of subsequent surface modifications.

Modification of the GCE with juglone (JUG/GCE) resulted in a substantial increase in the *R*_ct_ to 15.00 ± 0.05 kΩ, suggesting that the organic layer partially blocks the electrode surface and hinders electron transfer between the redox probe and the electrode. This conclusion is further supported by high value of Warburg impedance (σ = 25.0 ± 0.07 kΩ s^−1/2^), indicates that the transport of electroactive species across the modified interface is also impeded. The formation of the juglone film is further evidenced by the marked increase in the double-layer capacitance (*C*_dl_ = 57.92 ± 0.03 μF cm^−2^), reflecting changes in the interfacial structure and charge distribution following surface modification. The MC/GCE electrode exhibits a pronounced enlargement of the capacitive semicircle, corresponding to the highest charge transfer resistance (22.0 ± 0.04 kΩ), indicating the slowest electron transfer among all investigated electrodes. Although mesoporous carbon significantly increased the electroactive surface area (15.21 ± 0.03 mm^2^), the impedance data suggest that the porous layer introduces additional interfacial resistance, resulting in slower charge-transfer kinetics. This interpretation is supported by the lowest heterogeneous electron transfer rate constant (1.60 ± 0.01)·10^−6^ m s^−1^) obtained for the MC/GCE electrode. Therefore, while the mesoporous carbon enhances the available electroactive surface, its morphology appears to impede efficient electron transport between the redox probe and the underlying electrode.

Finally, the electrode modified with the nanocomposite of juglone and mesoporous carbon (JUG-MC/GCE) exhibited improved electrochemical characteristics compared with the individual modifiers. The lower charge transfer resistance (12.0 ± 0.05 kΩ) indicating that the combination of both materials facilitates electron transport across the electrode/electrolyte interface. In addition, the electroactive surface area remained high (13.04 ± 0.01 mm^2^), while the double-layer capacitance increased to 34.61 ± 0.03 μF cm^−2^, reflecting the beneficial contribution of juglone to the interfacial properties of the porous carbon layer. These findings suggest that the combination of juglone and mesoporous carbon produces a synergistic effect, balancing the large accessible surface area of the carbon material with improved charge-transfer characteristics at the electrode/electrolyte interface.

To assess the effect of electrode modification on the oxidation of melatonin, comparative DPV measurements were performed in the presence of 0.2 mg L^−1^ MEL in 0.1 mol L^−1^ McIlvaine buffer solution (pH 2.8) using the bare GCE and the modified electrodes (Figure 2B). Under these conditions, only a weak electrochemical response for the bare GCE was recorded. The MEL oxidation peak at approximately 0.80 V was barely distinguishable from the background current, indicating limited electrochemical activity of the unmodified electrode toward melatonin oxidation. Modification of the GCE with either juglone (JUG/GCE) or mesoporous carbon (MC/GCE) resulted in only a slight enhancement of the electrochemical response toward melatonin compared with the bare electrode. In both cases, a weak oxidation peak was observed at approximately 0.80 V, with the peak current only marginally exceeding the corresponding background signal. Although JUG may facilitate electron transfer and MC provides an increased electroactive surface area, neither modifier alone was sufficient to produce a significant improvement in the oxidation signal.

The enhanced analytical performance toward melatonin oxidation originates probably directly from the catalytic behavior of the juglone/mesoporous carbon composite. Acting as a solid-state redox mediator, the immobilized juglone undergoes a rapid, reversible two-electron/two-proton quinone/hydroquinone transformation. This redox-active interface provides an alternative, low-energy pathway that accelerates electron-transfer kinetics compared to unmodified glassy carbon electrodes. The mesoporous carbon support plays a crucial role by providing a high-surface-area network that maximizes the spatial distribution and accessibility of juglone molecules, while strong π-π interactions anchor them firmly within the matrix. Consequently, basing on our observations, this specific chemical-to-structural synergy lowers the activation overpotential, facilitating efficient charge percolation and resulting in the observed signal amplification for melatonin determination.

In contrast, the JUG-MC/GCE electrode exhibited a pronounced and well-defined oxidation peak centered at approximately 0.80 V vs. Ag|AgCl|3M KCl reference electrode. As shown in the inset of Figure 2B, the peak current was approximately 17-times higher than those obtained with the bare GCE, demonstrating the superior analytical performance of the composite electrode. The substantial increase in peak current observed for JUG-MC/GCE can be attributed to a synergistic effect between juglone and mesoporous carbon. The mesoporous carbon provides a high specific surface area and a porous structure that promotes adsorption and accumulation of melatonin molecules at the electrode interface. Simultaneously, juglone acts as a redox mediator, facilitating electron transfer and lowering the kinetic barrier of the oxidation process. The combination of these two components results in accelerated charge-transfer kinetics and a significantly amplified electrochemical response. Furthermore, comparison of the analytical and background voltammograms reveals that the enhanced signal observed for JUG-MC/GCE originates predominantly from melatonin oxidation rather than from an increase in capacitive current. The background current remains relatively unchanged, while a distinct faradaic peak appears in the presence of melatonin, indicating high sensitivity and favorable signal-to-background characteristics.

### 3.3. Electrochemical Behavior of Melatonin on JUG-MC/GCE

**Figure 3:**
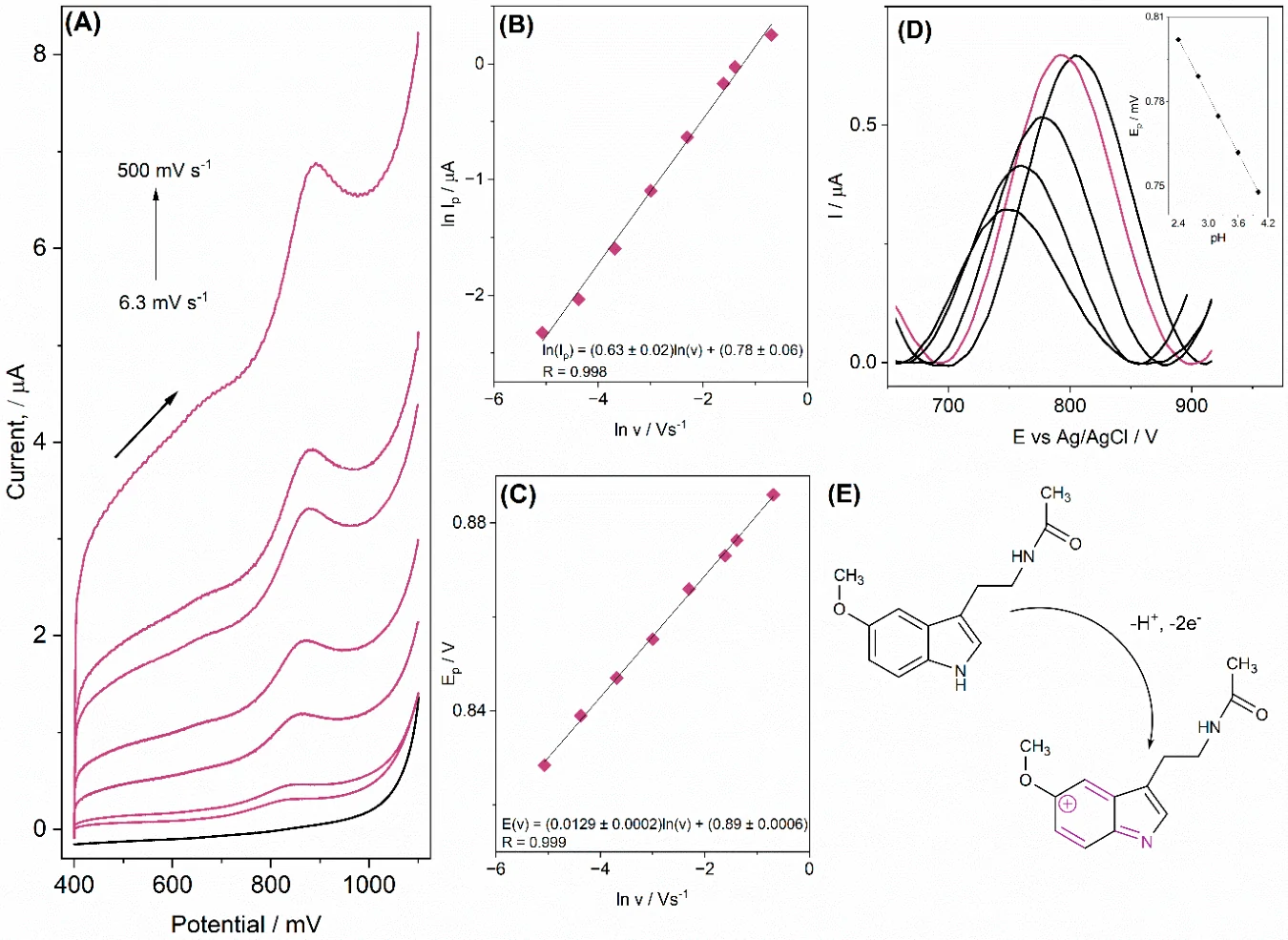
(**A**) CV curves recorded on JUG-MC/GCE for the increasing scan rate values in the range of 0.0063 to 0.5 V s^−1^ in the presence of 1 mg L^−1^ MEL in 0.1 mol L^−1^ McIlvaine buffer solution (pH 2.8). Violet lines represent the anodic scan, whereas the black one the cathodic scan. The relationship between (**B**) the natural logarithm of the MEL oxidation peak current (*I_p_*) and the natural logarithm of the scan rate (*v*), and (**C**) the oxidation MEL peak potential (*E_p_*) and the natural logarithm of the scan rate (*v*). (**D**) DPVs registered for the oxidation of 0.2 mg L^−1^ MEL in 0.1 mol L^−1^ McIlvaine buffer solution of pH ranging from 2.4 to 4.0. Inset: The dependence between the peak potential (*E_p_*) and pH of the supporting electrolyte. (**E**) Proposed mechanism of MEL electrooxidation on JUG-MC/GCE.

To the best of our knowledge, this study represents the first use of a glassy carbon electrode modified with a juglone–mesoporous carbon composite for the electrochemical determination of melatonin. To elucidate the electrooxidation behavior of melatonin at the JUG-MC/GCE interface, cyclic voltammetry was employed. The influence of the scan rate on the melatonin oxidation peak was evaluated over a broad range from 6.3 to 500 mV s^−1^ (Figure 3A). Notably, the complete absence of a corresponding reduction peak in the reverse scan confirms that the electrochemical oxidation of melatonin on this modified platform is an irreversible process. To gain deeper insights into the underlying reaction kinetics, the relationship between the voltammetric peak current and the scan rate parameters was analyzed. A linear correlation was established between the peak current and the square root of the scan rate. To further distinguish between diffusion, adsorption or kinetic controlled regimes, a double-logarithmic plot of peak current versus scan rate was constructed (Figure 3B). The resulting slope of 0.63 deviates from the theoretical value of 0.5 for purely diffusion-controlled processes, thereby confirming that the electro-oxidation of melatonin at the JUG-MC/GCE surface is governed by a mixed kinetic-diffusion control mechanism. To comprehensively map the electrochemical pathway of melatonin oxidation, CV was performed at varying scan rates, complemented by DPV across a range of pH conditions. The number of electrons participating in the rate-determining step of MEL oxidation was calculated from the linear dependence of the peak potential on the natural logarithm of the scan rate (Figure 3C). Utilizing the standard kinetic equation for irreversible processes:

(6) Ep=E0 + RTαnF0.780 + lnD012ks + lnαnFvRT12

and given the experimental slope of 0.0129, the electron transfer coefficient (*α*) was assumed to be 0.5. Consequently, the total number of exchanged electrons during the oxidation process was calculated to be 2. This result was further verified using the following equation:

(7) nα=0.048Ep−Ep12

which confirmed that two electrons were exchanged during MEL oxidation.

Furthermore, the influence of the medium’s acidity on the MEL oxidation mechanism was evaluated using a 0.1 mol L^−1^ McIlvaine buffer solution across a pH range of 2.4 to 4.0. As demonstrated in Figure 3D, both the peak current and peak potential of melatonin are highly pH-dependent. The optimum electrochemical response, characterized by maximum peak current, superior signal stability, and high reproducibility, was achieved at pH 2.8, which was consequently selected as the supporting electrolyte for all subsequent analytical experiments. As the pH of the buffer increased, the oxidation peak potential shifted systematically toward less positive values, indicating the direct participation of protons in the electrode reaction. The linear relationship between the peak potential and the solution pH is defined by the following regression equation:

(8) $$E_{p}=-0.034\;pH\;+\;0.88$$

The experimental slope of −0.034 V pH^−1^ implies a specific stoichiometric ratio where the number of electrons transferred is double the number of protons involved in the rate-determining step. This mechanistic deduction aligns seamlessly with previously documented literature regarding the electrochemical pathways of melatonin. The possible scheme of MEL electrooxidation on the JUG-MC/GCE surface is presented in Figure 3E. Initially, the melatonin molecule undergoes the loss of a proton from the indole ring (specifically at the pyrrole –N–H position) coupled with the abstraction of two electrons from the conjugated aromatic system. Consequently, this oxidation step converts the neutral parent molecule into a positively charged carbocation intermediate (+1 charge). The resulting charge is stabilized by extended π-electron delocalization across the bicyclic indole scaffold, as highlighted by the rearranged conjugated double bonds within the ring system. Throughout this initial electron transfer stage, both the methoxy moiety (–OCH_3_) at the C-5 position and the N-acetylaminoethyl side chain (–CH_2_CH_2_NHCOCH_3_) at the C-3 position remain structurally unreactive.

### 3.4. Optimization of Experimental Conditions

The effect of the experimental conditions on the oxidation response of melatonin was investigated by means of a univariate optimization approach using a 0.2 mg L^−1^ MEL solution in 0.1 mol L^−1^ McIlvaine buffer solution (pH 2.8). The DPV parameters were optimized by varying the step potential (*E_s_*, 1–6 mV), pulse amplitude (*dE*, ±10–60 mV), current sampling time (*t*_s_, 5–20 ms), and waiting time (*t*_w_, 5–20 ms). The optimal operating conditions were selected based on the peak current intensity, peak shape, symmetry, peak potential, and signal-to-noise ratio. Under these criteria, the most favorable analytical response was obtained using a step potential of 5 mV, a pulse amplitude of 50 mV, a sampling time of 10 ms, and a waiting time of 10 ms.

### 3.5. Analytical Performance

**Figure 4:**
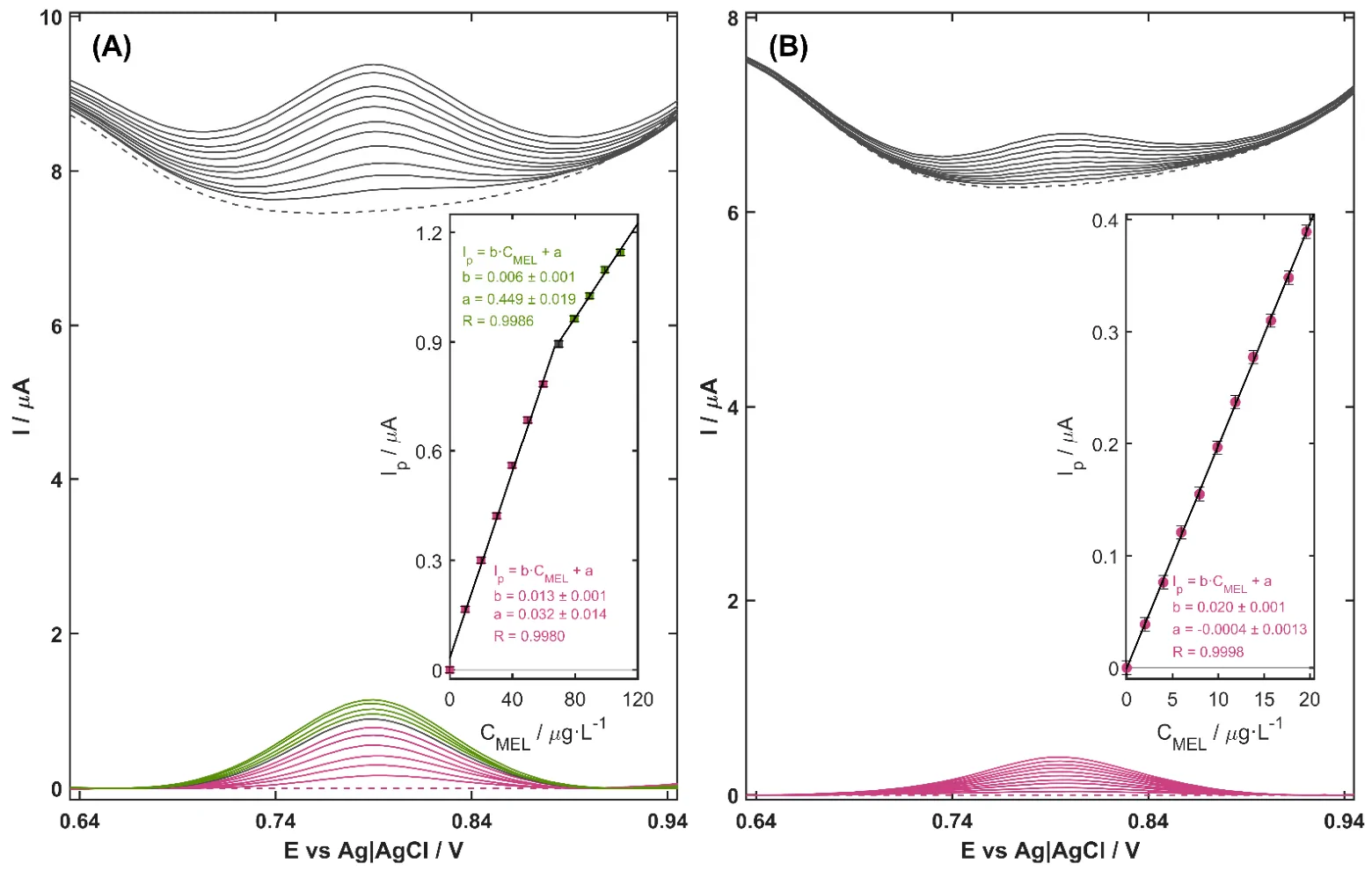
The DPV curves recorded on the JUG-MC/GCE for the increasing concentration of MEL in the range from blank (dashed line) to (**A**) 108.8 µg L^−1^, and (**B**) 19.6 µg L^−1^ (grey lines). The colored lines represent the voltammograms obtained after background correction. Inset: corresponding calibration plots. Experimental conditions as in Figure 2B.

To validate the developed protocol of melatonin determination with the use of JUG-MC/GCE, DP voltammograms for increasing concentration of MEL were recorded under the optimal conditions (Figure 4). The background current recorded in the supporting electrolyte (dashed line) was subtracted from each voltammogram to obtain the analytical response. The background-corrected peak currents were subsequently used to construct the calibration curves presented in Figure 4. The regression parameters, together with the analytical characteristics of the method, including the linear range (*LR*), limit of detection (*LOD*), and limit of quantification (*LOQ*), are listed in Table 2.

To ensure reliable quantification over a broad concentration range, calibration was performed using two MEL stock solutions (10 mg L^−1^ and 1 mg L^−1^), corresponding to the data shown in Figure 4A and Figure 4B, respectively. In both cases, the voltammetric response exhibited a well-defined and concentration-dependent increase in peak current, while the peak potential remained essentially invariant, confirming a stable and reproducible electrochemical process of melatonin oxidation at the JUG-MC/GCE. For the higher concentration range (Figure 4A), a broader calibration interval was established, in which the peak current showed a linear dependence on MEL concentration over two linear ranges: 9.9–69.5 µg L^−1^ and 69.5–108.8 µg L^−1^ with a correlation coefficient equal to 0.9980 and 0.9986, respectively. At the same time, the observed slight variation in slope between two calibration ranges may be associated with possible surface saturation effects and changes in mass transport and interfacial processes at higher analyte concentrations. In contrast, the low-concentration range (Figure 4B) allowed for an accurate quantification of MEL at trace levels (2.0–19.6 µg L^−1^) with enhanced sensitivity. The calculated limit of detection (LOD), which is equal to 0.21 µg L^−1^ (0.9 nM), confirms the suitability of the proposed method for the trace-level determination of MEL.

Both calibration sets confirm that the JUG-MC/GCE exhibits robust, stable, and reproducible electrochemical performance over a wide concentration range of MEL. The repeatability and short-term stability of the proposed sensor were evaluated using ten independently fabricated JUG-MC/GCE electrodes prepared according to the procedure described in Section 2.3. For each electrode, five consecutive DPV scans were recorded in 0.1 mol L^−1^ McIlvaine buffer solution (pH 2.8) containing MEL at concentrations of 80 μg L^−1^ and 8 μg L^−1^. The calculated relative standard deviation (RSD) of the oxidation peak current were 1.2% and 1.8%, respectively, confirming excellent repeatability and short-term operational stability of the sensor. The fabrication reproducibility was subsequently evaluated from the analytical responses of the ten independently prepared electrodes, providing an RSD of 11%, which demonstrates good reproducibility of the electrode preparation procedure and its analytical performance. The long-term stability of the sensor was assessment after storage under ambient laboratory conditions (22 ± 2 °C, in air, protected from direct light), with measurements performed after 7, 14, and 21 days. The changes in the oxidation peak current concentration remained did not exceed 17.4% for either MEL concentration, confirming satisfactory long-term storage stability of the JUG/MC-GCE.

To further demonstrate the significance of the proposed sensing platform, its analytical performance was compared with recently reported electrochemical sensors for melatonin determination (Table 3). As can be seen, a variety of advanced sensing strategies have recently been proposed, including molecularly imprinted polymers (MIPs) [27,28,29,30], graphene derivatives [24], conductive polymers [23], MXene-based nanocomposites [29], and cyclodextrin-functionalized electrodes [23]. Although these platforms generally exhibit excellent analytical performance, their fabrication often requires multistep synthesis procedures, sophisticated functionalization protocols, or specialized electrode materials. In contrast, the proposed JUG-MC/GCE is prepared by a simple drop-casting procedure using a conventional glassy carbon electrode and readily available modifier components. Despite this straightforward fabrication strategy, the developed sensor achieved an ultralow LOD, which is among the lowest values reported for electrochemical melatonin determination in recent years.

### 3.6. Interference Studies

The selectivity of the proposed JUG-MC/GCE was evaluated by examining the effect of different classes of potential interferents on the electrochemical oxidation signal of MEL. Electroactive species commonly encountered in biological matrices, including ascorbic acid and uric acid, as well as compounds frequently present in dietary supplements, such as glucose, caffeine, citric acid, starch, cellulose, titanium dioxide, and magnesium stearate, were investigated. The interference studies were carried out by separately adding glucose and caffeine (0.5 g L^−1^) to the supporting electrolyte containing 0.1 mg L^−1^ MEL, resulting in interferent-to-analyte concentration ratios ranging from 10:1 to 500:1. Similarly, the influence of ascorbic acid, citric acid, and uric acid on the electrochemical oxidation signal of MEL was investigated by gradually adding each interferent to the supporting electrolyte containing 0.1 mg L^−1^ MEL until a 100-fold excess of the interferent relative to the analyte was achieved. To assess the effect of starch, cellulose, titanium dioxide, and magnesium stearate on the electrochemical response of 0.1 mg L^−1^ MEL, the powdered excipients were individually introduced into the voltammetric cell in portions ranging from 3 to 9 mg. For each interferent, the percentage change in the peak current relative to the signal recorded in the absence of the interferent was calculated. An interference effect was considered to occur when the presence of the interferent caused a change in the MEL peak current exceeding ±10% of its initial value or resulted in a noticeable distortion of the peak shape and/or a shift in the peak potential.

Of all the tested interferents, only ascorbic acid and uric acid significantly affected the electrochemical response of MEL. A fourfold excess of ascorbic acid relative to MEL concentration resulted in an approximately 2-fold increase in the analytical signal. The pronounced enhancement of the analytical signal observed in the presence of ascorbic acid is most likely associated with its electrochemical oxidation at the JUG-MC/GCE electrode, leading to a partial overlap of the oxidation currents of ascorbic acid and melatonin. In the case of uric acid, a slight suppression of the MEL signal was observed, with the peak current decreasing to 83% of its initial value in the presence of a 20-fold excess of the interferent relative to MEL. This effect may be associated with competitive adsorption phenomena at the electrode surface or partial surface fouling caused by the oxidation products of uric acid. In contrast, none of the investigated pharmaceutical excipients produced significant interference under the tested conditions. These findings indicate that the proposed JUG-MC/GCE sensor provides satisfactory selectivity for the determination of melatonin in commercial pharmaceutical formulations and dietary supplements.

### 3.7. Real Sample Analysis

The applicability of the proposed analytical method was evaluated by determining melatonin in commercially available pharmaceutical formulation and dietary supplements in different dosage forms, including tablets, powder, and jellybeans. Following the sample preparation procedure outlined in Section 2.5, all samples were subsequently analyzed using the standard addition method. For each sample, three independent determinations were performed. The analytical results are reported as mean ± SD. The agreement between the experimentally determined melatonin contents and the values declared by the manufacturers was evaluated by calculating the relative error (RE), while the precision of the proposed method was assessed using the RSD.

**Figure 5:**
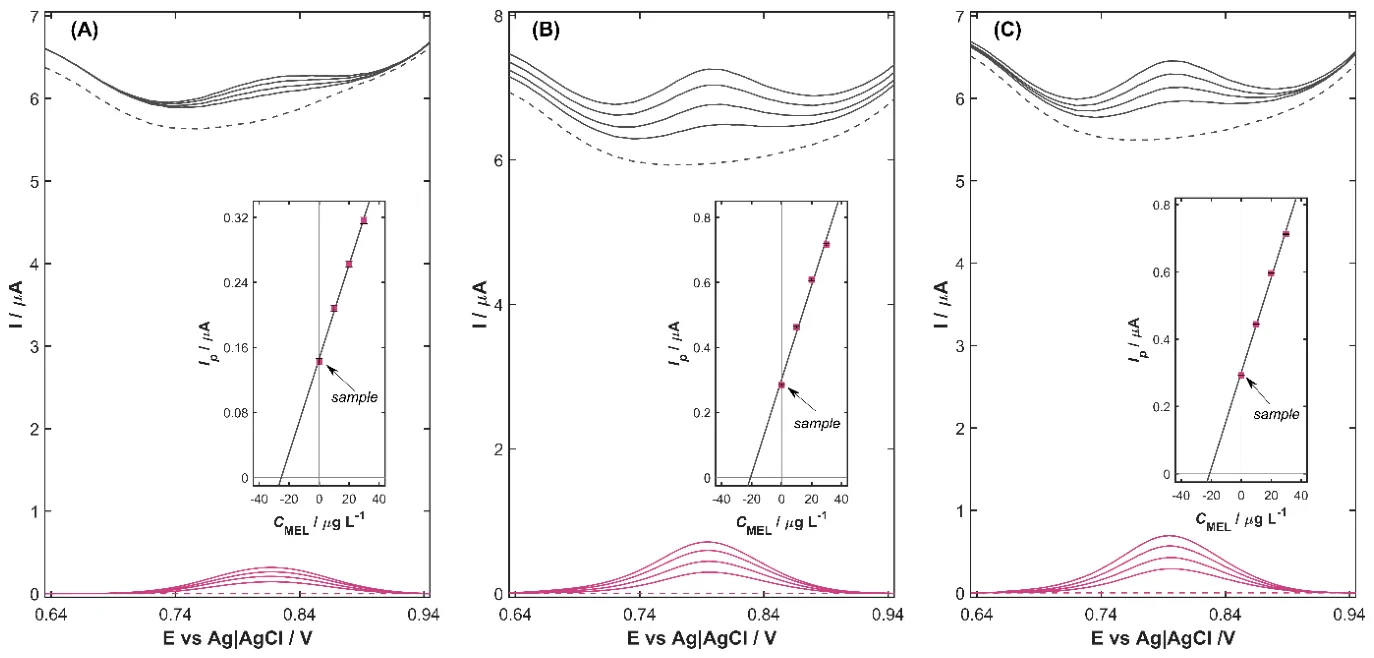
Investigation of real samples: (**A**) Pharmaceutical formulation, (**B**) Dietary supplement (tablet), and (**C**) Dietary supplement (powder). The experimental voltammograms are shown in grey, whereas the background-corrected curves—in color. Inset: corresponding calibration plots. Experimental conditions are as in Figure 2B.

The voltammograms recorded for the analyzed samples (Figure 5) a well-defined oxidation peak corresponding to melatonin was observed at the expected potential. The peak shape remained symmetrical and well resolved, indicating that the sample matrices did not introduce significant interference with the electrochemical response of JUG-MC/GCE. Moreover, no additional peaks overlapping with the melatonin signal were detected, demonstrating the satisfactory selectivity of the proposed method toward MEL determination in complex commercial products.

The determined MEL contents (Table 4) were in good agreement with the values declared by the manufacturers. The RE ranged from −3.0% to 6.0%, indicating method applicability in commercial samples, including pharmaceutical formulations and dietary supplements with different matrices. The low RSD values obtained for samples in the form of tablets and capsules further confirmed the good precision and repeatability of the proposed analytical procedure, demonstrating its suitability for the routine quality control of commercially available melatonin-containing products. While the RSD value for jelly candies sample (14.4%) reflects a moderate dispersion, it is considered acceptable for complex dietary supplement matrix containing gelatin or pectin based compounds. The inherent heterogeneity of the jelly matrix, combined with the challenges of achieving complete sample homogenization and quantitative extraction of the analyte from the sticky polymer network, typically accounts for such variations in precision. Nevertheless, the nominal value of 1 mg falls well within the calculated confidence interval, confirming the accuracy of the developed method.

## 4. Conclusions

Herein, we report the development and electrochemical validation of a novel juglone/mesoporous carbon modified GCE tailored for the quantification of melatonin in commercial pharmaceuticals and dietary supplements. The combination of the redox-active quinone moieties of juglone with the porous carbon-based structure of mesoporous carbon creates a composite modifying layer that provide complementary electrochemical functionalities. The presence of juglone contribute to redox mediation, while mesoporous carbon facilitate the formation of a conductive framework, collectively contributing to the improved electrochemical response of the modified electrode. Consequently, the developed sensor enable reliable electrochemical determination of melatonin in dietary supplements samples, with satisfactory selectivity in the presence of common matrix components. This sensing platform operates with an extensive linear dynamic range and achieves a nanomolar detection limit without requiring any prior accumulation or preconcentration steps. Given its economical fabrication protocol, robust long-term stability, and reproducible operation in intricate sample matrices, the proposed electrode represents a highly viable tool for routine quality assurance in pharmaceutical analysis.

## Figures and Tables

**Table 1:** The comparison of electrochemical properties of tested electrodes achieved from EIS measurements (*n* = 3).

Electrode	Parameter						
	*R*_ct_ /kΩ	*σ* /kΩ s^−1/2^	*A*_el_ /mm^2^	*C*_dl_ /µF cm^−2^	*k*_s_ /m s^−1^	RMES (*Z’*) /kΩ	RMES (−*Z″*) /kΩ
GCE	1.38 ± 0.02	3.50 ± 0.08	8.04 ± 0.03	27.43 ± 0.02	(4.81 ± 0.06)·10^−5^	0.7	0.5
JUG/GCE	15.00 ± 0.05	25.00 ± 0.07	1.12 ± 0.02	57.92 ± 0.03	(3.16 ± 0.04)·10^−5^	4.1	2.4
MC/GCE	22.00 ± 0.04	1.85 ± 0.09	15.21 ±0.03	1.13 ± 0.01	(1.60 ± 0.01)·10^−6^	0.5	0.3
JUG-MC/GCE	12.00 ± 0.05	2.16 ± 0.04	13.04 ± 0.01	34.61 ± 0.03	(3.42 ± 0.01)·10^−6^	0.2	0.1

**Table 2:** Summary of analytical parameters for melatonin determination.

Parameter	Unit	Figure 4A	Figure 4B
LR	µg L^−1^	9.9–69.5 69.5–108.8	2.0–19.6
Intercept *a*	µA	0.032 ± 0.014	−0.0004 ± 0.0013
		0.449 ± 0.019	
Slope *b*	µA L µg^−1^	0.013 ± 0.001	0.020 ± 0.001
		0.006 ±0.001	
*R*	-	0.9980	0.9998
		0.9986	
LOD	µg L^−1^ (nM)	3.6 (15.5)	0.21 (0.9)
LOQ	µg L^−1^ (nM)	10.9 (46.9)	0.65 (2.8)

*LOD* = 3.3*SD*/*b*, and *LOQ* = 10*SD*/*b*, where *SD*: the standard deviation of y-intercepts of the regression line, and *b*: the slope of the calibration curve.

**Table 3:** Comparison of recently reported electrochemical sensors of melatonin.

Electrode	Technique	LR/µM	LOD/µM	Samples	Ref.
^1^ CPE	SWV	0.5–80	0.083	food, pharmaceutical	[20]
^2^ GC/MoS_2_/CB[8]	DPV	1.25–50.0	0.380	pharmaceutical, human urine and serum	[26]
^3^ MIP/GCE	DPV	0.2–200	0.061	urine, saliva	[28]
^4^ α-Fe_2_O_3_/C–Fe-gC_3_N_4_/SPCE	DPV	0.1–233.1	0.004	tablet, human urine, tart cherries	[31]
^5^ HD-CNT-fiber cross-section microelectrode	SWV	0.05–100	0.010	food, urine, pharmaceutical	[22]
^6^ MIPs/4-AMP/PEDOT/GCE	LSV	0–100	0.171	capsules	[27]
^7^ MIP-PTBO/MWCNTs/GCE	DPV	1–1000	0.027	human serum, tablets	[30]
^8^ MIP/Cu_x_O/MXene/SPCE	DPV	5–700	0.029	human urine	[29]
^9^ PGMGCPE	CV	4–30	0.73	pharmaceutical	[25]
^10^ PDPC/GCE	DPV	0.010–850.0	0.00 52	human serum and urine	[23]
^11^ g-C_3_N_4_-GNPs-MWCNT/GCE	DPV	0.5–10	0.0092	human plasma	[24]
^12^ ZnOFs/HpβCD@rGOs/GCE	DPV	0.014–0.149 1.149–643.341	0.0105	human serum	[21]
^13^ poly(BPB)-modified GCE	SWV	0.1–40.0	0.0299	tablet, human serum, urine	[19]
^14^ JUG-MWCNT/CPE	DPV	0.002–0.16	0.021	tablets, dietary supplements	[32]
^15^ JUG-MC/GCE	DPV	0.009–0.47	0.0009	tablets, dietary supplements	This work

^1^ CPE—unmodified carbon paste electrode; ^2^ MoS_2_—nanosheets and cucurbit[8]uri modified GCE; ^3^ MIP/GCE—methylene blue molecularly imprinted polymer modified GCE; ^4^ α-Fe_2_O_3_/C–Fe-gC_3_N_4_/SPCE—porous hematite embedded C and Fe codoped graphitic carbon nitride screen printed carbon electrode; ^5^ HD-CNT—highly densified carbon nanotube fibers cross-section microelectrode; ^6^ MIPs/4-AMP/PEDOT/GCE—molecular imprinting 4-aminothiophenol/3,4-ethylenedioxythiophene modified GCE; ^7^ MIP-PTBO/MWCNTs/GCE—molecularly imprinted polytoluidine blue O (and multi-walled carbon nanotubes modified GCE; ^8^ MIP/Cu_x_O/MXene/SPCE—Cu*_x_*O/MXene modified SPCE, ^9^ PGMGCPE—poly (glycine)-modified graphite CPE; ^10^ PDPC/GCE—poly(vinyl alcohol),dextran, polypyrrole. copper oxide particles modified GCE; ^11^ g-C_3_N_4_-GNPs-MWCNT/GCE—graphitic carbon nitride-gold nanoparticles-multi-walled carbon nanotubes modified GCE; ^12^ ZnOFs/HpβCD@rGOs/GCE—zinc oxide flaky structures/hydroxypropyl-beta-cyclodextrin functionalized reduced graphene oxide sheets modified GCE; ^13^ poly(BPB)-modified GCE—bromophenol blue polymer modified GCE; ^14^ JUG-MWCNT/CPE—juglone and functionalized multiwalled carbon nanotubes modified CPE; ^15^ JUG-MC/GCE – juglone and mesoporous carbon modified GCE; SWV—Square Ware Voltammetry; LSV—Linear Sweep Voltammetry.

**Table 4:** Results of melatonin determination in pharmaceutical formulation and dietary supplements.

Sample	Amount of MEL/mg per Tablet		*RE* [%]	*RSD* [%]
	Declared	Found $\bar{x}$ ± *SD*		
Pharmaceutical formulation (tablet)	3.0	3.16 ± 0.11	5.1	3.5
Dietary supplement (tablet)	1.0	1.04 ± 0.09	4.0	8.6
Dietary supplement (capsule)	1.0	1.06 ± 0.06	6.0	5.7
Dietary supplement (jelly candies)	1.0	0.97 ± 0.14	−3.0	14.4

$\bar{x}$*—*mean value; *SD*—standard deviation; *RSD%* = *SD/*$\bar{x}$·100%.

## Data Availability

The raw data supporting the conclusions of this article will be made available by the authors on request.
